# Response to High‐Dose Vitamin D Supplementation Is Specific to Imaging Modality and Skeletal Site

**DOI:** 10.1002/jbm4.10615

**Published:** 2022-03-08

**Authors:** Lauren A Burt, Leigh Gabel, Emma O Billington, David A Hanley, Steven K Boyd

**Affiliations:** ^1^ McCaig Institute for Bone and Joint Health, Cumming School of Medicine University of Calgary Calgary Canada

**Keywords:** AGING, BONE DENSITY, BONE QCT/ΜCT, DUAL‐ENERGY X‐RAY ABSORPTIOMETRY, HIGH‐RESOLUTION PERIPHERAL QUANTITATIVE COMPUTED TOMOGRAPHY, MICROARCHITECTURE, OSTEOPOROSIS, PTH/VIT D/FGF23, VITAMIN D

## Abstract

High‐dose vitamin D supplementation (4000 or 10,000 IU/d) in vitamin D‐sufficient individuals results in a dose‐dependent decrease in radius and tibia total bone mineral density (Tt.BMD) compared with 400 IU/d. This exploratory analysis examined whether the response to high‐dose vitamin D supplementation depends on imaging modality and skeletal site. Participants were aged 55 to 70 years, not osteoporotic, with serum 25(OH)D 30 to 125 nM. Participants’ radius and tibia were scanned on high‐resolution peripheral quantitative computed tomography (HR‐pQCT) to measure Tt.BMD, trabecular bone volume fraction (Tb.BV/TV), trabecular separation (Tb.Sp), cortical thickness (Ct.Th), and finite element analysis (FEA) estimated failure load. Three‐dimensional image registration was used. Dual‐energy X‐ray absorptiometry (DXA) scans of the hip, spine, and radius measured areal BMD (aBMD) and trabecular bone score (TBS). Constrained linear mixed‐effects models determined treatment group‐by‐time and treatment group‐by‐time‐by‐sex interactions. The treatment group‐by‐time interaction previously observed for radial Tt.BMD was observed at both ultradistal (UD, *p* < 0.001) and 33% (*p* < 0.001) aBMD sites. However, the treatment group‐by‐time‐by‐sex interaction observed for radial Tt.BMD was not observed with aBMD at either the UD or 33% site, and the 4000 and 400 groups did not differ. Registered radial FEA results mirrored Tt.BMD. An increase in Tb.Sp and decrease in Ct.Th underpinned dose‐dependent changes in radial BMD and strength. We observed no effects in DXA‐based aBMD at the hip or spine or TBS. At the tibia, we observed a time‐by‐treatment group effect for Tb.BV/TV. Given that DXA measures at the radius did not detect sex differences or differences between the 4000 and 400 groups, HR‐pQCT at the radius may be more sensitive for examining bone changes after vitamin D supplementation. Although DXA did not reveal treatment effects at the hip or spine, whether that is a true skeletal site difference or a lack of modality sensitivity remains unclear. © 2022 The Authors. *JBMR Plus* published by Wiley Periodicals LLC on behalf of American Society for Bone and Mineral Research.

## 1 Introduction

For decades, vitamin D supplementation has been prescribed for bone health, particularly in postmenopausal females, and has been extensively reviewed for its effects on fracture, falls, and bone mineral density (BMD).^(^ ^1^ ^)^ The results from a meta‐analysis indicate in the absence of vitamin D deficiency, vitamin D supplementation has no clinically meaningful effect on BMD, regardless of dose.^(^ ^1^ ^)^ These findings were established using dual‐energy X‐ray absorptiometry (DXA) to assess areal BMD.

A potentially more sensitive technique used to assess bone health is high‐resolution peripheral quantitative computed tomography (HR‐pQCT). This technique assesses compartment‐specific volumetric BMD and bone microarchitecture at the peripheral skeleton, most commonly the distal radius and tibia.^(^ ^2^ ^)^ Accompanied with finite element analysis (FEA) to estimate bone strength, the clinical implementation of HR‐pQCT is expanding. Although it is common for studies to use both DXA and HR‐pQCT, it is rare to have longitudinal data on more than one imaging modality employed at the same skeletal site so that direct comparisons are possible.

Recently, HR‐pQCT has been used to explore changes in total volumetric BMD (Tt.BMD) and bone strength after 3 years of high‐dose vitamin D_3_ supplementation in healthy males and females who were vitamin D‐sufficient and without osteoporosis at baseline.^(^ ^3^ ^)^ Supplementation with either 10,000 IU or 4000 IU per day resulted in larger Tt.BMD losses compared with 400 IU per day at both the radius and tibia.^(^ ^4^ ^)^ Further analyses revealed decreases in Tt.BMD were driven by females, whereas changes in Tt.BMD in males did not reach significance.^(^ ^5^ ^)^ However, direct comparisons between imaging modalities (ie, HR‐pQCT versus DXA) and skeletal sites (ie, tibia, radius, hip, spine) have not been reported. Therefore, the objective of this exploratory analysis was to determine if the response to high‐dose vitamin D supplementation is dependent on imaging modality and skeletal site.

## 2 Materials and Methods

### 2.1 Study design

Our double‐blind clinical trial investigated the effects of high‐dose daily vitamin D supplementation on bone density and strength over 3 years^(^ ^3^ ^)^ (Clinical Trial Registration NCT01900860). The trial was conducted between August 2013 and December 2017.

### 2.2 Participants

As reported previously, 373 individuals met the inclusion criteria and were randomized, with 311 forming the main cohort.^(^ ^4^ ^)^ This exploratory analysis focuses on the main cohort, consistent with our previous publications.^(^ ^4^, ^5^ ^)^ Recruitment and randomization occurred over 15 months, ending in November 2014. Participants were males and females, aged 55 to 70 years, with DXA (lumbar spine and total hip) areal BMD *T*‐scores above −2.5 SD, serum 25‐hydroxyvitamin D [25(OH)D] between 30 and 125 nM, and normal serum calcium. Participants were excluded if their screening serum 25(OH)D was lower than 30 nM or above 125 nM; serum calcium was lower than 2.10 mM or above 2.55 mM; or daily vitamin D supplement use exceeded 2000 IU within the past 6 months. Additional exclusion criteria have been published previously.^(^ ^4^ ^)^ The trial was approved by the Conjoint Health Research Ethics Board at the University of Calgary, and Health Canada provided a letter of No Objection. Each participant provided written informed consent before study participation and an independent Data Safety and Monitoring Board oversaw the study.

### 2.3 Randomization and intervention

Participants were randomized, within sex, in a 1:1:1 ratio to receive 400, 4000, or 10,000 IU vitamin D_3_ cholecalciferol, taken orally once per day in the form of liquid drops (Ddrops, Woodbridge, Canada): five drops/d (400: 80 IU/drop; 4000: 800 IU/drop; or 10,000: 2000 IU/drop). As described previously,^(^ ^3^, ^4^ ^)^ this was a double‐blind study where the lowest dose (400 IU daily) was chosen with the assumption that participants would receive at least 200 IU/d from diet and sunlight exposure, therefore, receiving the recommended dietary allowance (RDA) of vitamin D (600 IU/d).^(^ ^6^ ^)^ Adherence was calculated from daily diaries as number of days of vitamin D administration versus total number of days of follow‐up, expressed as a percentage.

Unfortunately, unbeknownst to the investigators until after the conclusion of the trial, the laboratory source of the 2000 IU/drop preparation was changed during the study, and the new preparation was less stable than the preparations used in the initial years of the study. This resulted in a decline in mean serum 25(OH)D levels in the 10,000 IU/d treatment group, affecting the 24‐ and 36‐month measurements.

Calcium intake was assessed by a food‐frequency questionnaire.^(^ ^7^ ^)^ Participants not consuming the recommended dietary allowance of calcium (1200 mg/d)^(^ ^8^ ^)^ received calcium citrate tablets (each containing 300 mg elemental calcium) as needed up to a maximum of 600 mg/d to approximate a total daily intake of 1200 mg.

### 2.4 Outcomes and procedures

The primary and secondary outcome variables of the trial have been reported previously^(^ ^4^, ^5^ ^)^ and include variables collected using HR‐pQCT, FEA, and DXA, as well as physical function and quality of life variables. Specifically, primary variables were total volumetric BMD (Tt.BMD) and bone strength (failure load estimated by FEA) at the distal radius and tibia. The secondary bone outcomes for the trial included total hip areal BMD by DXA, HR‐pQCT measures of cortical and trabecular volumetric BMD (Ct.BMD, Tb.BMD), cortical porosity (Ct.Po) and trabecular number (Tb.N), assessment of balance (postural sway), physical function (timed up‐and‐go and grip strength) and quality of life (SF36 questionnaire). For current analyses, skeletal imaging variables not previously presented as primary or secondary variables will be explored.

Participants’ non‐dominant radius and left tibia were assessed with HR‐pQCT (nominal isotropic voxel of 60.7 μm, XtremeCT II, Scanco Medical, Bruttisellen, Switzerland), following three‐dimensional image registration^(^ ^9^ ^)^ at baseline, 6, 12, 24, and 36 months. At each site, standard variables including trabecular bone volume fraction (Tb.BV/TV, %), trabecular thickness (Tb.Th, mm), trabecular separation (Tb.Sp, mm) and cortical thickness (Ct.Th, mm) were measured. In addition, cortical pore diameter (Ct.Po.Dm, mm), cortical perimeter (Ct.Pm, mm), connectivity density (Conn.D, 1/mm^3^),^(^ ^10^ ^)^ degree of anisotropy (DA, unitless), and structural model index (SMI, unitless)^(^ ^11^ ^)^ were assessed. Scans were assessed for motion^(^ ^12^ ^)^ and percent overlap between registered images. Scans with motions scores of four or higher and scans where percent overlap was below 75% were removed from the analysis. Our FEA bone strength estimates of failure load employed our newly developed three‐dimensional registration technique.^(^ ^13^ ^)^ We transformed the boundary conditions with the 4 × 4 transforms resulting from the HR‐pQCT registration, and thus the registered volumes have non‐parallel proximal and distal surfaces and a non‐uniform thickness. An axial compression test was simulated on each scan using a 1% compressive strain, Young's modulus of 8748 MPa, and a Poisson's ratio of 0.3 (FAIM, v8.0, Numerics88 Solutions, Calgary, Canada).^(^ ^14^ ^)^

DXA scans of the spine, hip, and radius occurred annually (GE Lunar iDXA, GE Healthcare, Madison, WI, USA; enCORE v16), measuring areal BMD at the lumbar spine (LS), femoral neck (FN), and radius (ultradistal [UD] and 33% sites). For participants with a body mass index (BMI) >15 and <37, trabecular bone score (TBS; iNsight, v3.0) was calculated at the spine using the same vertebrae as the areal BMD analysis. Absolute 10‐year risk of major osteoporotic fracture and hip fracture were calculated using the Canadian Fracture Risk Assessment (FRAX) tool, with inclusion of femoral neck areal BMD.^(^ ^15^ ^)^

HR‐pQCT precision in our laboratory for three‐dimensional registered images is <1% for density and <2.9% for microarchitecture parameters, with the exception of cortical porosity (<11.8%).^(^ ^9^ ^)^ Our registered FEA assessment of failure load has a CV RMS of <1%.^(^ ^13^ ^)^ DXA precision ranges from 0.51% to 1.14%.

### 2.5 Statistical analysis

The primary aim of our original trial was to investigate the effect of high doses of vitamin D supplementation (4000 IU/d or 10,000 IU/d) on bone density and strength assessed by HR‐pQCT and FEA,^(^ ^3^ ^)^ with the 400 IU/d group representing the RDA intake or reference group (supplementation: 400 IU; diet: 200 IU). Our sample size calculation has been previously published.^(^ ^4^ ^)^

Participant characteristics were described using means and standard deviations (continuous data) or total number and percentage (categorical data). Individuals who received at least one dose of the study drug and at least one follow‐up visit were included in this analysis. We analyzed outcome variables using constrained linear mixed‐effects models, as in previous work. Fixed effects included treatment group, sex, and a quadratic effect of time if warranted. Random effects included time in which both intercept and slope could vary. Missing data were accounted for using the mixed‐effects model.^(^ ^16^ ^)^ Specifically, we explored time‐by‐treatment group interactions, which, if significant, indicated a significant treatment group effect.^(^ ^4^ ^)^ Time‐by‐treatment group‐by‐sex interactions were investigated if a significant time‐by‐treatment group interaction was reported. Significant time‐by‐treatment group‐by‐sex interactions indicated the treatment group effect differed by sex.^(^ ^5^ ^)^ We were not powered to explore time‐by‐treatment group‐by‐age interactions. Analyses were conducted using Stata (V.16, StataCorp, College Station, TX, USA) and significance was defined as *p* < 0.05.

## 3 Results

As previously reported, 311 participants (53% male) were randomized and enrolled in the study.^(^ ^5^ ^)^ Ninety‐two percent of participants completed the trial, and supplementation adherence was >99% in all groups. Eight participants did not have one or more follow‐up assessments and were excluded from the analysis. A total of 303 participants were included in this exploratory analysis (Table 1). {TBL 1} Average age was 62.1 (±4.2 SD) years, BMI 27.7 (±4.6 SD) kg/m^2^, and 95% of the cohort were White. Specifically, males were 176.4 (±6.7 SD) cm tall and weighed 85.4 (±11.9 SD) kg. Females were 162.7 (±6.1 SD) cm tall and weighed 72.9 (±15.2 SD) kg. Additional participant characteristics, split by sex, have been published.^(^ ^5^ ^)^ At baseline, no individuals were vitamin D deficient (25OHD <30 nM), 7.6% of the cohort were classified as vitamin D insufficient (25OHD 30–50 nM), and 92.4% were vitamin D sufficient (25OHD >50 nM). Ten‐year FRAX risk for any major osteoporotic fracture was 5.7% (±2.5 SD) at baseline and 6.5% (±3.0 SD) at study end. FRAX risk of hip fracture was 0.5% (±0.5 SD) and 0.8% (±0.9 SD) at baseline and 36 months, respectively. Participant characteristics, split by sex, have been reported elsewhere.^(^ ^5^ ^)^

**Table 1:** Participant Characteristics at Baseline

	400 IU	4000 IU	10,000 IU
	*n* = 105	*n* = 97	*n* = 101
	Mean (SD)	Mean (SD)	Mean (SD)
Age (years)	62.2 (4.2)	62.6 (4.4)	61.9 (4.1)
Height (m)	1.7 (0.1)	1.7 (0.1)	1.7 (0.1)
Weight (kg)	81.6 (14.8)	80.7 (15.4)	77.6 (14.4)
Serum 25(OH)D (nM)	76.7 (21.1)	81.3 (20.1)	78.4 (18.4)
FRAX any major fracture	6.0 (2.8)	5.5 (1.9)	5.9 (2.5)
FRAX hip fracture	0.5 (0.5)	0.5 (0.6)	0.5 (0.5)
DXA			
Lumbar spine aBMD (g/cm^2^)	1.183 (0.172)	1.202 (0.161)	1.188 (0.176)
Lumbar spine *T*‐score	0.046 (1.433)	0.203 (1.334)	0.062 (1.432)
Total hip aBMD (g/cm^2^)	1.022 (0.138)	1.035 (0.140)	1.008 (0.138)
Total hip *T*‐score	0.116 (1.093)	0.224 (1.109)	0.000 (1.097)
Femoral neck aBMD (g/cm^2^)	0.965 (0.131)	0.972 (0.124)	0.951 (0.132)
Femoral neck *T*‐score	−0.525 (0.937)	−0.471 (0.899)	−0.626 (0.953)
HR‐pQCT (mg HA/cm^3^)			
Radius Tt.BMD	324.9 (61.5)	335.9 (65.3)	329.7 (60.0)
Radius Ct.BMD	887.6 (50.0)	899.2 (51.3)	904.0 (53.3)
Radius Tb.BMD	163.1 (40.3)	160.1 (39.9)	155.9 (40.2)
Tibia Tt.BMD	301.2 (58.3)	314.1 (52.9)	306.5 (52.6)
Tibia Ct.BMD	853.9 (61.5)	868.6 (52.9)	871.5 (59.0)
Tibia Tb.BMD	176.4 (37.7)	174.8 (35.2)	171.9 (38.7)

aBMD = areal bone mineral density; Tt.BMD = total bone mineral density; Ct.BMD = cortical bone mineral density; Tb.BMD = trabecular bone mineral density.

For HR‐pQCT analyses, 25 participants had at least one radius scan removed due to motion or inadequate overlap (<75%) of registered images. Two individuals had all radius scans removed from analyses. For DXA, 42 individuals had all LS scans removed due to degenerative changes making the spine clinically unreportable, and an additional 18 individuals had TBS at the spine removed due to BMI >37.^(^ ^17^ ^)^ No individuals had BMI <15. One individual had all hip scans removed due to degenerative changes making the hip clinically unreportable, and another individual had one DXA radius scan removed because of an artifact in the region of interest.

### 3.1 Time‐by‐treatment group interactions

**Fig. 1:**
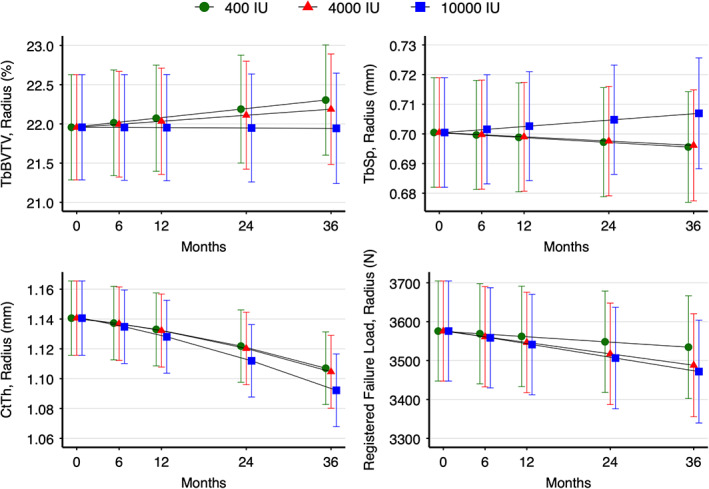
Trabecular bone volume fraction (Tb.BV/TV), trabecular separation (Tb.Sp), cortical thickness (Ct.Th), and bone strength (failure load) at the radius over 3 years of vitamin D supplementation, by treatment group. The modeled data show the mean 95% confidence intervals (CIs) for the predicted values. Green circles = 400 IU group; red triangles = 4000 IU group; blue squares = 10,000 IU group.

There were significant time‐by‐treatment group interactions at the radius for several HR‐pQCT variables (Fig. 1). {FIG1} Based on the mixed‐model analysis, at trial end, Tb.BV/TV was lower for the 10,000 IU group compared with the 400 IU group (−0.36%; 95% confidence interval [CI] −0.60 to −0.12), with no difference between the 4000 IU and 400 IU groups (−0.11%; 95% CI −0.36 to 0.13). This is equivalent to changes from the mean baseline values of 1.6% (400 IU), 1.1% (4000 IU), and −0.1% (10,000 IU) for radius Tb.BV/TV. Tb.Sp was greater in the 10,000 IU group compared with the 400 IU group (0.01 mm; 95% CI 0.00 to 0.02), with no difference between the 4000 and 400 IU groups (0.00 mm; 95% CI −0.01 to 0.01). Changes from the mean baseline values were equivalent to −0.7% (400 IU), −0.6% (4000 IU), and 0.9% (10,000 IU) for radius Tb.Sp. Ct.Th was lower for the 10,000 IU group compared with the 400 IU group (−0.01 mm; 95% CI −0.03 to −0.00), with no difference between the 4000 IU and 400 IU groups (−0.00 mm; 95% CI −0.01 to 0.01). Changes from the mean baseline values were equivalent to −2.9% (400 IU), −3.2% (4000 IU), and −4.2% (10,000 IU) for radius Ct.Th. Registered failure load was lower for the 10,000 IU group (−62.8 N; 95% CI −101.7 to −23.9) and 4000 IU (−46.5 N; 95% CI −85.8 to −7.2) compared with the 400 IU group. Changes from the mean baseline values were equivalent to 1.2% (400 IU), −2.5% (4000 IU), and −2.9% (10,000 IU) for registered failure load at the radius.

For DA, the differences between the 10,000 IU group and the 4000 IU group compared with the 400 IU group were not statistically significant (10,000: 0.01 [unitless]; 95% CI −0.00 to 0.02, and 4000: −0.01 [unitless]; 95% CI −0.02 to 0.00); however, DA significantly declined over time in the 400 IU and 4000 IU groups but not the 10,000 IU group. Changes from the mean baseline values were equivalent to −0.7% (400 IU), −1.4% (4000 IU), and 0.1% (10,000 IU) for radius DA. Percent change for radius HR‐pQCT variables are presented in Table 2{TBL 2} and raw radius data across all time points in Supplemental Table S1.

**Table 2:** Baseline, 3‐Year, and Percent Change for HR‐pQCT Variables at the Radius

	Baseline	36 months	Change
	Mean (SD)	Mean (SD)	%
400 IU			
Tb.BV/TV (%)	22.50 (6.01)	22.79 (6.26)	1.58
Tb.Th (mm)	0.23 (0.02)	0.23 (0.02)	1.01
Tb.Sp (mm)	0.68 (0.12)	0.68 (0.13)	−0.70
Ct.Th (mm)	1.12 (0.2)	1.09 (0.19)	−2.94
Ct.Pm (mm)	72.92 (9.41)	74.85 (9.93)	1.86
Ct.Po.Dm (mm)	0.20 (0.03)	0.21 (0.03)	1.10
DA (unitless)	1.42 (0.08)	1.40 (0.08)	−0.70
Conn.D (1/mm3)	2.84 (0.67)	2.86 (0.67)	0.12
SMI (unitless)	2.95 (1.24)	2.92 (1.18)	−3.59
Failure load (*N*)^a^	3673.66 (1160.84)	3640.9 (1170.95)	−1.16
4000 IU			
Tb.BV/TV (%)	21.92 (6.03)	22.16 (6.22)	1.05
Tb.Th (mm)	0.23 (0.02)	0.24 (0.02)	0.86
Tb.Sp (mm)	0.69 (0.13)	0.69 (0.13)	−0.62
Ct.Th (mm)	1.16 (0.23)	1.14 (0.22)	−3.15
Ct.Pm (mm)	70.60 (8.43)	72.87 (8.69)	2.09
Ct.Po.Dm (mm)	0.21 (0.03)	0.21 (0.03)	2.13
DA (unitless)	1.40 (0.07)	1.38 (0.06)	−1.43
Conn.D (1/mm^3^)	2.78 (0.07)	2.78 (0.67)	−0.08
SMI (unitless)	3.29 (1.49)	3.24 (1.32)	−2.93
Failure load (*N*)^a^	3544.88 (1128.94)	3503.44 (1127.21)	**−2.46**
10,000 IU			
Tb.BV/TV (%)	21.45 (5.78)	21.39 (5.76)	**−0.06**
Tb.Th (mm)	0.23 (0.02)	0.24 (0.02)	0.45
Tb.Sp (mm)	0.73 (0.21)	0.74 (0.2)	**0.92**
Ct.Th (mm)	1.13 (0.22)	1.08 (0.22)	**−4.24**
Ct.Pm (mm)	70.53 (9.27)	72.13 (9.83)	1.91
Ct.Po.Dm (mm)	0.21 (0.04)	0.21 (0.04)	1.94
DA (unitless)	1.40 (0.09)	1.41 (0.08)	0.06
Conn.D (1/mm^3^)	2.71 (0.70)	2.65 (0.67)	−2.03
SMI (unitless)	3.30 (1.38)	3.19 (1.22)	−2.99
Failure load (*N*)^a^	3511.10 (1131.24)	3377.10 (1176.20)	**−2.91**

Tb.BV/TV = trabecular bone volume fraction; Tb.Th = trabecular thickness; Tb.Sp = trabecular separation; Ct.Th = cortical thickness; Ct.Pm = cortical perimeter; Ct.Po.Dm = cortical pore diameter; DA = degree of anisotropy; Conn.D = connectivity density; SMI = structural model index.

^a^ Registered failure load. Bold values indicate significantly different from the 400 IU group (*p* < 0.05). Percent change values are from the constrained linear mixed‐effects models.

At the tibia, the only time‐by‐treatment group effect observed was for Tb.BV/TV, where the 10,000 IU group was lower than the 400 IU group (−0.20%; 95% CI −0.37 to −0.03), with no difference between the 4000 IU and 400 IU groups (0.02%; 95% CI −0.16 to 0.19). Changes from the mean baseline values were equivalent to 2.1% (400 IU), 2.1% (4000 IU), and 1.3% (10,000 IU) for tibia Tb.BV/TV. Percent change for tibia HR‐pQCT variables are presented in Supplemental Table S2 and raw tibia data across all time points in Supplemental Table S3.

Time‐by‐treatment group interactions were not statistically significant for Tb.Th (radius and tibia), Tb.Sp (tibia), Ct.Th (tibia), Ct.Po.Dm (radius and tibia), Ct.Pm (radius and tibia), Conn.D (radius and tibia, although there was a trend *p* = 0.08 at the radius where the 10,000 IU group decreased over time and the 4000 and 400 IU groups did not), DA (tibia), SMI (radius and tibia), and registered failure load (tibia).

DXA results revealed time‐by‐treatment group interactions at the radius. Based on the mixed‐model analysis, at trial end UD areal BMD was lower for the 10,000 IU group compared with the 400 IU group (−0.011 g/cm^2^; 95% CI −0.016 to −0.006), with no difference between the 4000 IU and 400 IU groups (−0.001 g/cm^2^; 95% CI −0.006 to 0.004). This is equivalent to changes from the mean baseline values of 0.8% (400 IU), −1.1% (4000 IU), and −3.1% (10,000 IU) for UD areal BMD. Similar findings were observed for the 33% areal BMD with lower values for the 10,000 IU group compared with the 400 IU group (−0.015 g/cm^2^; 95% CI −0.022 to −0.007), with no difference between the 4000 IU and 400 IU groups (−0.003 g/cm^2^; 95% CI −0.011 to 0.005). This is equivalent to changes from the mean baseline values of 0.1% (400 IU), −0.2% (4000 IU), and − 1.5% (10,000 IU) for 33% areal BMD.

Time‐by‐treatment group interactions were not statistically significant for LS, TH areal BMD, or TBS (*p* > 0.05). Percent change for DXA variables are presented in Table 3{TBL 3} and raw DXA data across all time points in Supplemental Table S4.

**Table 3:** Baseline, 3‐Year, and Percent Change for DXA Variables

	Baseline	36 months	Change
	Mean (SD)	Mean (SD)	%
400 IU			
LS aBMD (g/cm^2^)	1.183 (0.172)	1.184 (0.181)	−0.04
LS TBS	1.397 (0.096)	1.402 (0.103)	0.15
FN aBMD (g/cm^2^)	0.965 (0.131)	0.953 (0.134)	−1.36
TH aBMD (g/cm^2^)	1.022 (0.138)	1.015 (0.141)	−1.08
UD aBMD (g/cm^2^)	0.482 (0.098)	0.481 (0.100)	−0.84
33% aBMD (g/cm^2^)	0.906 (0.138)	0.908 (0.145)	0.10
4000 IU			
LS aBMD (g/cm^2^)	1.202 (0.161)	1.205 (0.182)	0.28
LS TBS	1.375 (0.100)	1.390 (0.087)	0.57
FN aBMD (g/cm^2^)	0.972 (0.124)	0.967 (0.134)	−0.71
TH aBMD (g/cm^2^)	1.035 (0.140)	1.030 (0.149)	−0.74
UD aBMD (g/cm^2^)	0.476 (0.130)	0.473 (0.102)	−1.08
33% aBMD (g/cm^2^)	0.908 (0.130)	0.906 (0.139)	−0.24
10,000 IU			
LS aBMD (g/cm^2^)	1.188 (0.176)	1.186 (0.192)	−0.76
LS TBS	1.413 (0.095)	1.411 (0.093)	−0.42
FN aBMD (g/cm^2^)	0.951 (0.132)	0.943 (0.140)	−0.69
TH aBMD (g/cm^2^)	1.008 (0.138)	1.001 (0.139)	−0.63
UD aBMD (g/cm^2^)	0.471 (0.103)	0.457 (0.106)	**−3.07**
33% aBMD (g/cm^2^)	0.909 (0.128)	0.894 (0.146)	**−1.51**

aBMD = areal bone mineral density; LS = lumbar spine; TBS = trabecular bone score; FN = femoral neck; TH = total hip; UD = ultradistal.

Bold values indicate significantly different from the 400 IU group (*p* < 0.05). Percent change values are from the constrained linear mixed‐effects models.

### 3.2 Time‐by‐treatment group‐by‐sex interactions

**Fig. 2:**
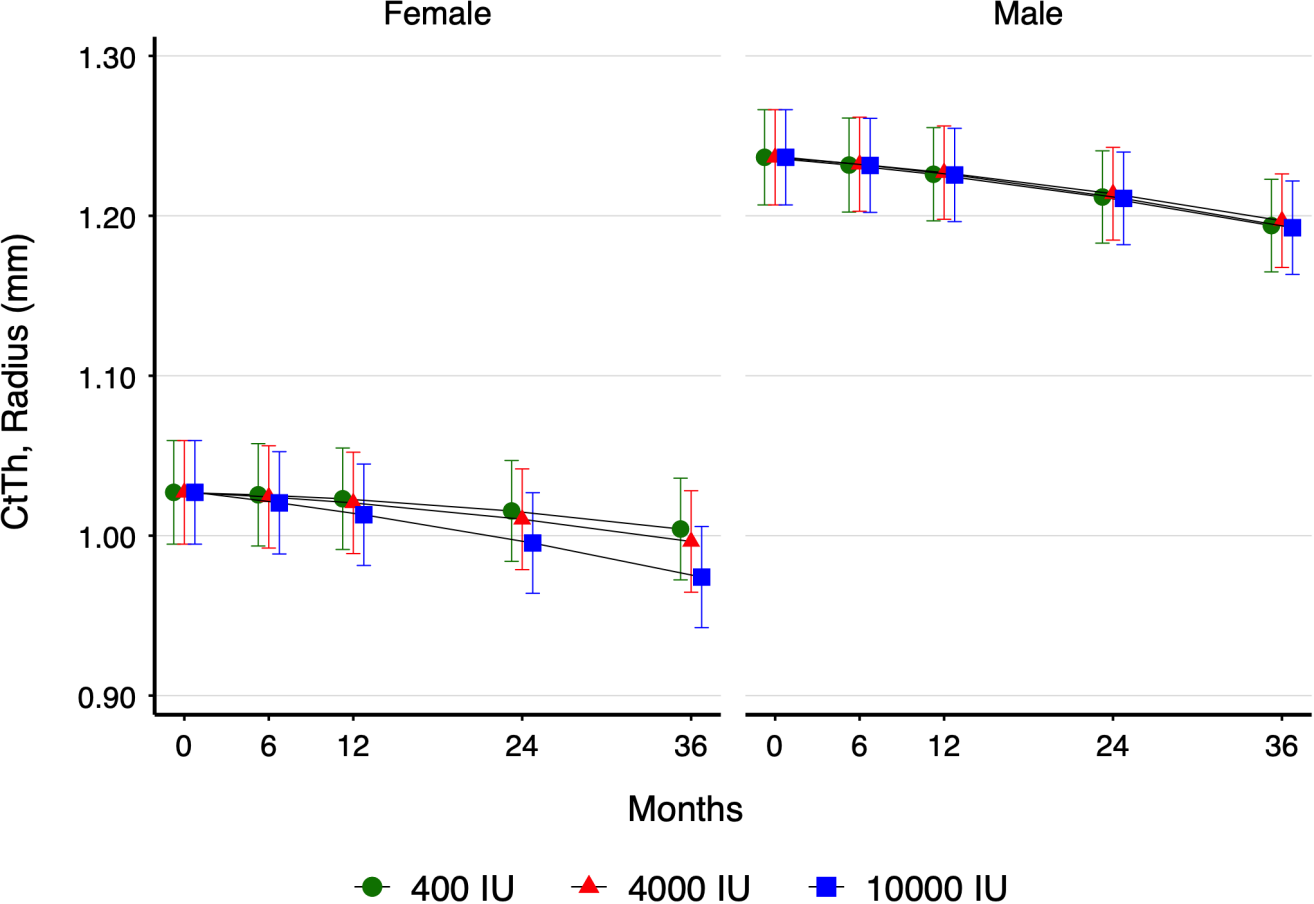
Sex differences in cortical thickness (Ct.Th) at the radius over 3 years of vitamin D supplementation, by treatment group. Females are shown on the left and males on the right. Green circles = 400 IU group; red triangles = 4000 IU group; blue squares = 10,000 IU group.

At the radius, the only time‐by‐treatment group‐by‐sex interaction observed was for Ct.Th where females in the 10,000 IU group had lower Ct.Th than females in the 400 IU group (Fig. 2). {FIG2} Based on the mixed‐effects models, at trial end for females there was a difference between the 10,000 IU and 400 group IU (−0.03 mm; 95% CI −0.05 to −0.01), whereas there was no difference between the 4000 IU and 400 IU groups (−0.01 mm; 95% CI −0.03 to 0.01). This is equivalent to changes from the mean baseline values of −2.2% (400 IU), −3.0% (4000 IU), and − 5.2% (10,000 IU) for females. For males, no dose‐dependent effect was observed; however, all groups declined over time: −3.5% (400 IU), −3.2% (4000 IU), and −3.6% (10,000 IU).

At the tibia, no time‐by‐treatment group‐by‐sex interactions were observed (*p* > 0.05). Similarly, time‐by‐treatment group‐by‐sex interactions were not observed for DXA variables (*p* > 0.05).

## 4 Discussion

This exploratory analysis assessed whether the skeletal response to 3 years of high‐dose vitamin D supplementation is dependent on imaging modality and skeletal site. We found that previously reported Tt.BMD changes^(^ ^4^ ^)^ are underpinned by microarchitectural adaptations captured in the trabecular compartment (Tb.BV/TV, Tb.Sp) and the cortex (Ct.Th). Furthermore, we showed significant changes to bone strength using registered FEA that were not previously detected.^(^ ^4^ ^)^ Interestingly, at the radius, some of the changes observed with HR‐pQCT were also detected with DXA areal BMD, but DXA did not have sufficient sensitivity to observe sex‐based differences or dose‐dependent differences between the 4000 and 400 IU daily treatment groups. Examining skeletal sites, both radius and tibia showed time‐by‐treatment group interactions using HR‐pQCT, but other skeletal sites such as hip and spine did not detect similar interactions after vitamin D supplementation using DXA. The conundrum is whether the absence of observed interactions at the hip and spine is due to the lower sensitivity of DXA or a true lack of effect of vitamin D supplementation at these skeletal sites. Given that we showed at the radius that DXA had lower sensitivity than HR‐pQCT, it is possible that changes at the hip and spine are occurring after vitamin D supplementation but not at a level that can be detected by DXA.

Few trials employ multiple modalities at the same skeletal site, so this is an opportunity to compare the findings at the distal radius measured by both HR‐pQCT and DXA. In our previous primary analysis using HR‐pQCT, daily high‐dose vitamin D supplementation of 10,000 and 4000 IU resulted in greater decreases in Tt.BMD at the radius compared with 400 IU,^(^ ^4^ ^)^ whereby the negative dose‐response relationship was driven by females in the study.^(^ ^5^ ^)^ DXA radius scans at the ultradistal site overlap with the HR‐pQCT scan region,^(^ ^18^ ^)^ and so we expected similar results for both modalities. In cross‐sectional studies, moderate to strong correlations (0.48–0.75) have been reported for Tt.BMD measurements from HR‐pQCT compared with ultradistal forearm areal BMD by DXA,^(^ ^19^ ^)^ and we confirm similar correlations (*r* = 0.48 for Tt.BMD and 33% radial areal BMD; *r* = 0.67 for Tt.BMD and UD radial areal BMD). Three‐year changes in the 10,000 IU group were similar between DXA and HR‐pQCT, with observations of −3.1% areal BMD at the ultradistal radius via DXA and −3.5% for Tt.BMD with HR‐pQCT.^(^ ^4^ ^)^ Although both modalities showed similar changes in the 10,000 IU group, the ability to detect differences among treatment groups and sex was different in this 3‐year longitudinal study, suggesting a difference in modality sensitivity. Like HR‐pQCT, using DXA we observed greater decreases in areal BMD in the 10,000 IU group compared with the 400 IU group at both the ultradistal and 33% radius sites. However, we did not observe differences between the 4000 and 400 IU groups that were evident in Tt.BMD with HR‐pQCT.^(^ ^4^ ^)^ Furthermore, using DXA we did not observe a significant sex effect where females lost more bone compared with males, as we identified with HR‐pQCT.^(^ ^5^ ^)^ A possible reason for the lack of sex differences observed using DXA may be the smaller bone size of females, whereby the slight bone loss observed due to supplementation may be more challenging for DXA to detect in smaller bones compared with larger bones. In longitudinal studies, precisely estimating change is important, and our findings suggest that HR‐pQCT coupled with three‐dimensional image registration has increased sensitivity compared with DXA measured at the same skeletal site.

Most trials are limited to a short period of observation because of practical limitations (ie, cost, study participant burden); therefore maximizing the ability to detect change, even if it is small, is important because these changes may manifest into substantial effects over long periods of time (eg, vitamin D supplementation over decades). In an ancillary study from the Vitamin D and Omega‐3 Trial (VITAL) where 2‐year change in bone health in osteoporotic individuals was explored,^(^ ^20^, ^21^ ^)^ no time‐by‐treatment group and time‐by‐treatment group‐by‐sex effect on areal BMD after vitamin D supplementation was reported. The VITAL study used both DXA and peripheral quantitative computed tomography (pQCT; lower resolution than HR‐pQCT), but did not scan the radius on DXA. It is possible that their use of a lower daily supplementation dose (2000 IU), in a population that was not vitamin D deficient at study entry, might not result in skeletal effects, but it is also possible that smaller dose‐related effects were not detectable by their assessment methods. The challenge with concluding whether there are skeletal site differences associated with vitamin D supplementation is that the sensitivity to these changes is dependent on imaging modality. Because neither HR‐pQCT nor pQCT can be employed at sites like the hip and spine, it is possible that these sites are affected in the VITAL study and ours, albeit below the detectable threshold of the modality.

In the current study, we observed several microarchitectural changes after high‐dose vitamin D supplementation that underpin our previous Tt.BMD findings. Microarchitectural changes at the radius were not limited to one bone compartment, as both cortical and trabecular bone significantly changed over time, particularly in the 10,000 IU group. Our previously reported decline in radial cortical BMD^(^ ^4^ ^)^ appears to be driven by thinning of the cortex, as cortical thickness declined in all supplementation groups over time. Furthermore, radial cortical thickness was the only variable to show a sex effect where decreases for females in the 10,000 IU group were larger than those in the 400 IU group. It is possible that high doses of vitamin D amplify the effects of menopause on bone turnover and bone loss, resulting in the increased cortical bone loss observed in females in this study. Alternatively, since females are smaller than males on average, a dose of 10,000 IU for all individuals might mean female participants were receiving a relatively greater vitamin D exposure.

A fundamental shift in the radius trabecular microarchitecture seems to occur because of large daily doses of vitamin D. The increase in trabecular separation we observed in the 10,000 IU group is well aligned with our previously described decreases in trabecular number,^(^ ^4^ ^)^ suggesting small trabeculae were lost. The trend for decreased trabecular connectivity over time in the 10,000 IU group supports the notion that the deterioration of trabeculae is indicative of disconnections of rod‐like trabeculae.^(^ ^10^ ^)^ The degree of anisotropy decreased over 3 years, becoming more isotropic in both the 4000 and 400 IU groups but remaining constant in the 10,000 IU group. A possible explanation is that in the high‐dose group (10,000 IU) the loss of trabecular connections is accompanied by thinning of the remaining trabecular structure, leading to anisotropy remaining constant.

Using HR‐pQCT, we observed more prominent changes at the radius than the tibia after high‐dose vitamin D supplementation. We found changes in trabecular microarchitecture (Tb.BV/TV) at the tibia, but did not detect changes in cortical microarchitecture. Because the radius is a non‐weight‐bearing bone, it is possible that it is more sensitive to vitamin D supplementation compared with the tibia, where physical activity–associated loading plays a larger role.

Our previous publication reported a non‐significant trend for decreased bone strength, assessed with FEA, at the radius for the 10,000 and 4000 IU groups compared with the 400 IU group.^(^ ^4^ ^)^ At the time of that publication, we did not have a method to account for three‐dimensional image registration and therefore there was an inherent lack of reproducibility. Since then, we have developed an approach for coupling FEA with registration.^(^ ^13^ ^)^ We now show significantly lower radial bone strength in the 10,000 and 4000 IU groups, which mirrors registered total and cortical BMD findings at the radius, and is consistent with the trend in bone strength we previously reported. The reason unregistered finite element analysis has been the standard approach in the past is due to the technical challenges of applying axial compression boundary conditions to the non‐parallel surfaces that result from three‐dimensional registration. Our new technique uses the full common region from the three‐dimensional registration and the same region of bone is assessed at each time point, enhancing the sensitivity of the finite element method to capture changes in bone strength.

The results of our study should be interpreted in the context of some limitations. First, this was an exploratory analysis of our clinical trial; therefore, our original study design may be underpowered to detect sex and treatment group interactions. Second, it is worth noting that we previously reported the degradation of the vitamin D supplementation administered to the 10,000 IU group over the second half of the trial,^(^ ^4^ ^)^ possibly attenuating the 3‐year skeletal changes observed in this group. Third, participants in our trial were healthy and non‐osteoporotic. It is possible that participants with osteoporosis or who were vitamin D deficient would have responded differently. Finally, while we lacked statistical power to explore time‐by‐treatment group‐by‐age interactions, we have previously reported age‐related differences in Tt.BMD where younger females (ie, <65 years) lost more bone than older females.^(^ ^5^ ^)^

The modest decreases in BMD (areal BMD and Tt.BMD), microarchitecture, and strength were not associated with increased fracture risk in this study, which may raise questions about their clinical significance. However, it should be noted that the participants in this study were vitamin D sufficient and had normal bone density. In older individuals with osteoporosis, these observed effects of high‐dose vitamin D might be enough to have an impact on fracture risk.^(^ ^22^ ^)^

A novelty of our study is demonstrating, in an interventional study, that imaging modality can play a role in detecting significant interactions among variables, because we could compare measurements at the same skeletal site. DXA areal BMD measures at the radius did not detect sex differences or differences between the 4000 and 400 IU groups previously reported with HR‐pQCT.^(^ ^4^, ^5^ ^)^ Therefore, for the same skeletal site, HR‐pQCT was more sensitive than DXA in detecting a dose‐response effect of vitamin D supplementation, specifically a decrease in radial BMD. The tibia was less affected than the radius as only BMD^(^ ^4^, ^5^ ^)^ and Tb.BV/TV observed dose‐response changes by HR‐pQCT. At other skeletal sites, we did not observe dose‐response changes in DXA‐based areal BMD at the hip or spine, or TBS, although it is possible that the changes were too small to be detected by DXA. Detecting small negative effects of high‐dose vitamin D supplementation on the skeleton is important, as long‐term supplementation could build to more substantial skeletal effects.

## Disclosures

All authors state that they have no conflicts of interest.

## Data Sharing

The authors commit to making anonymized data that support the findings of this study available upon reasonable request. To gain access, data requestors will need to sign a data access agreement.

5

### Peer Review

The peer review history for this article is available at https://publons.com/publon/10.1002/jbm4.10615.

## Supporting information


**Supplemental Table S1.** Raw HR‐pQCT Results for the Radius at Each Time Point
**Supplemental Table S2.** Baseline, 3‐Year, and Percent Change for HR‐pQCT Variables at the Tibia
**Supplemental Table S3.** Raw HR‐pQCT Results for the Tibia at Each Time Point
**Supplemental Table S4.** Raw DXA Results at Each Time Point
