# Supplementary material for: Response to High‐Dose Vitamin D Supplementation Is Specific to Imaging Modality and Skeletal Site
Source: JBMR Plus. 2022 Mar 8;6(5):e10615. doi: 10.1002/jbm4.10615 (PMC9059471; doi:10.1002/jbm4.10615)
Supplement: Supplementary file 1 — Supplemental Table S1. Raw HR‐pQCT Results for the Radius at Each Time Point Supplemental Table S2. Baseline, 3‐Year, and Percent Change for HR‐pQCT Variables at the Tibia Supplemental Table S3. Raw HR‐pQCT Results for the Tibia at Each Time Point Supplemental Table S4. Raw DXA Results at Each Time Point [file JBM4-6-e10615-s001.pdf]

Supplementary Table 1. Raw HR-pQCT results for the radius at each time point

		0			6			12			24			36		
	**IU**	**N**	**Mean**	**SD**	**N**	**Mean**	**SD**	**N**	**Mean**	**SD**	**N**	**Mean**	**SD**	**N**	**Mean**	**SD**
**TtBMD**	400	104	324.91	61.5	102	325.73	61.80	103	323.23	61.11	98	322.99	61.77	99	320.15	61.11
	4000	96	335.89	65.26	94	334.62	65.11	94	333.09	65.41	92	330.44	66.18	90	328.64	66.08
	10000	99	329.71	60.00	97	327.39	60.34	97	324.70	59.75	97	320.86	60.15	93	317.31	61.68
**CtBMD**	400	104	887.55	50.02	102	884.85	49.92	103	882.20	51.18	98	882.21	49.38	99	879.6	50.99
	4000	96	899.20	51.29	94	897.15	49.39	94	890.43	51.14	92	889.18	52.06	90	886.26	52.43
	10000	99	903.97	53.33	97	899.34	52.67	97	891.89	53.48	97	889.80	53.16	93	884.93	54.16
**TbBMD**	400	104	163.06	40.32	102	164.36	40.55	103	164.37	41.30	98	165.18	42.09	99	164.77	42.49
	4000	96	160.41	39.93	94	159.91	39.80	94	160.90	40.27	92	160.89	41.58	90	161.49	42.00
	10000	99	155.94	40.17	97	155.69	40.31	97	156.11	40.30	97	155.43	40.80	93	155.15	40.13
**TbBV/TV**	400	104	22.50	6.01	102	22.55	6.11	103	22.67	6.13	98	22.78	6.26	99	22.79	6.26
	4000	96	21.92	6.03	94	21.81	6.03	94	21.99	6.05	92	22.08	6.19	90	22.16	6.22
	10000	99	21.45	5.78	97	21.36	5.82	97	21.44	5.80	97	21.47	5.82	93	21.39	5.76
**TbTh**	400	104	0.233	0.018	102	0.234	0.018	103	0.233	0.019	98	0.235	0.021	99	0.235	0.020
	4000	96	0.233	0.020	94	0.233	0.020	94	0.232	0.019	92	0.234	0.020	90	0.235	0.021
	10000	99	0.234	0.019	97	0.233	0.019	97	0.234	0.020	97	0.235	0.020	93	0.236	0.020
**TbSp**	400	104	0.68	0.12	102	0.68	0.14	103	0.68	0.13	98	0.68	0.13	99	0.68	0.13
	4000	96	0.69	0.13	94	0.69	0.13	94	0.69	0.14	92	0.69	0.13	90	0.69	0.13
	10000	99	0.73	0.21	97	0.73	0.21	97	0.73	0.22	97	0.73	0.21	93	0.74	0.20
**TbN**	400	104	1.41	0.20	102	1.43	0.22	103	1.43	0.22	98	1.43	0.21	99	1.43	0.21
	4000	96	1.41	0.21	94	1.43	0.22	94	1.42	0.21	92	1.41	0.21	90	1.43	0.21
	10000	99	1.37	0.25	97	1.37	0.25	97	1.37	0.25	97	1.36	0.24	93	1.36	0.25
**CtTh**	400	104	1.12	0.20	102	1.13	0.20	103	1.11	0.20	98	1.11	0.19	99	1.09	0.19
	4000	96	1.16	0.23	94	1.17	0.23	94	1.16	0.23	92	1.15	0.23	90	1.14	0.22
	10000	99	1.13	0.22	97	1.13	0.22	97	1.12	0.22	97	1.10	0.22	93	1.08	0.22

	400	104	1.00	0.56	102	0.98	0.61	103	1.02	0.63	98	1.02	0.65	99	1.04	0.64
**CtPo**	4000	96	0.87	0.45	94	0.87	0.46	94	0.98	0.58	92	0.97	0.55	90	0.97	0.53
	10000	99	0.88	0.59	97	0.92	0.63	97	0.95	0.64	97	0.98	0.65	93	1.00	0.63
	400	104	1.42	0.08	102	1.40	0.07	103	1.41	0.08	98	1.40	0.08	99	1.40	0.08
**DA**	4000	96	1.40	0.07	94	1.39	0.07	94	1.39	0.08	92	1.39	0.07	90	1.38	0.06
	10000	99	1.40	0.09	97	1.40	0.08	97	1.40	0.08	97	1.41	0.09	93	1.41	0.08
	400	104	2.84	0.67	102	2.84	0.69	103	2.87	0.68	98	2.81	0.67	99	2.86	0.67
**ConnD**	4000	96	2.78	0.68	94	2.79	0.69	94	2.79	0.69	92	2.76	0.70	90	2.78	0.67
	10000	99	2.71	0.70	97	2.71	0.71	97	2.71	0.69	97	2.69	0.69	93	2.65	0.67
	400	104	2.95	1.24	102	3.04	1.29	103	2.95	1.24	98	2.91	1.22	99	2.92	1.18
**SMI**	4000	96	3.29	1.49	94	3.36	1.32	94	3.22	1.40	92	3.15	1.34	90	3.24	1.32
	10000	99	3.30	1.38	97	3.34	1.40	97	3.24	1.30	97	3.17	1.38	93	3.19	1.22
	400	104	2700	1020	102	2669	1025	103	2688	989	98	2672	1015	99	2694	1022
**Failure Load**	4000	96	2580	990	94	2554	1005	94	2599	1012	92	2577	991	90	2550	994
	10000	99	2556	964	97	2550	1001	97	2517	988	97	2512	1013	93	2470	1001
	400	104	3673	1160	102	3646	1154	103	3667	1146	98	3654	1169	99	3640	1170
**Failure Load [a]**	4000	96	3544	1128	94	3520	1129	94	3562	1124	92	3532	1146	90	3503	1127
	10000	99	3511	1131	97	3478	1168	97	3462	1159	97	3439	1170	93	3377	1176

Trabecular bone volume fraction (TbBV/TV, %), trabecular thickness (TbTh, mm), trabecular separation (TbSp, mm), cortical thickness (CtTh, mm), cortical perimeter (CtPm, mm), cortical pore diameter (CtPoDm, mm), degree of anisotropy (DA, unitless), connectivity density (ConnD, 1/mm^3), structural model index (SMI, unitless). Failure load (N) is reported as unregistered (previously reported) and registered [a].

Supplementary Table 2. Baseline, three year and percent change for HR-pQCT variables at the tibia.

	Baseline Mean (SD)	36 months Mean (SD)	Change %
**400 IU**			
TbBV/TV (%)	25.46 (5.3)	25.9 (5.61)	2.07
TbTh (mm)	0.27 (0.04)	0.27 (0.03)	-0.09
TbSp (mm)	0.74 (0.12)	0.72 (0.12)	-1.69
CtTh (mm)	1.58 (0.36)	1.53 (0.32)	-3.33
CtPm (mm)	106.48 (10.35)	110.38 (11.4)	2.47
CtPoDm (mm)	0.24 (0.03)	0.23 (0.03)	-0.71
DA (unitless)	1.47 (0.09)	1.45 (0.09)	-1.13
ConnD (1/mm3)	2.57 (0.64)	2.65 (0.68)	2.35
SMI (unitless)	1.70 (0.98)	1.74 (0.93)	-0.30
Failure Load (N) [a]	9601.58 (2679.91)	9546.75 (2729.44)	-0.81
**4,000 IU**			
TbBV/TV (%)	25.15 (4.89)	25.63 (5.36)	2.14
TbTh (mm)	0.26 (0.03)	0.27 (0.03)	1.14
TbSp (mm)	0.74 (0.12)	0.73 (0.14)	-1.12
CtTh (mm)	1.66 (0.32)	1.59 (0.28)	-3.42
CtPm (mm)	102.77 (9.81)	106.78 (10.63)	2.81
CtPoDm (mm)	0.24 (0.03)	0.24 (0.04)	-0.76
DA (unitless)	1.45 (0.09)	1.43 (0.09)	-0.98
ConnD (1/mm3)	2.59 (0.09)	2.61 (0.61)	1.09
SMI (unitless)	1.90 (1.04)	1.93 (1.06)	0.17
Failure Load (N) [a]	9431.37 (2307.18)	9324.10 (2430.94)	-0.95
**10,000 IU**			
TbBV/TV (%)	24.91 (5.31)	25.39 (5.56)	**1.28**
TbTh (mm)	0.27 (0.02)	0.27 (0.03)	1.06
TbSp (mm)	0.77 (0.18)	0.77 (0.19)	-0.76
CtTh (mm)	1.60 (0.31)	1.54 (0.29)	-3.89
CtPm (mm)	103.86 (11.07)	107.44 (11.92)	2.64
CtPoDm (mm)	0.24 (0.03)	0.24 (0.03)	-1.10
DA (unitless)	1.45 (0.08)	1.44 (0.09)	-0.87
ConnD (1/mm3)	2.50 (0.71)	2.51 (0.73)	0.31
SMI (unitless)	1.86 (1.02)	1.80 (0.96)	-0.80
Failure Load (N)[a]	9303.04 (2480.59)	9164.83 (2557.39)	-1.54

[a] Registered failure load. Bold values indicate significantly different from the 400 IU group. Percent change values are from the constrained linear mixed effects models.

Trabecular bone volume fraction (TbBV/TV), trabecular thickness (TbTh), trabecular separation (TbSp), cortical thickness (CtTh), cortical perimeter (CtPm), cortical pore diameter (CtPoDm), degree of anisotropy (DA), connectivity density (ConnD), structural model index (SMI).

Supplementary Table 3. Raw HR-pQCT results for the tibia at each time point

		0			6			12			24			36		
	**IU**	**N**	**Mean**	**SD**	**N**	**Mean**	**SD**	**N**	**Mean**	**SD**	**N**	**Mean**	**SD**	**N**	**Mean**	**SD**
**TtBMD**	400	105	301.23	58.33	105	302.43	58.39	104	301.59	58.92	100	300.09	58.43	100	299.06	58.66
	4000	97	314.08	52.89	96	314.29	54.02	95	312.23	53.41	93	310.30	53.60	93	309.08	54.82
	10000	101	306.46	52.63	101	306.38	52.29	100	305.15	52.67	99	303.12	53.66	96	301.45	54.61
**CtBMD**	400	105	853.88	61.52	105	851.86	63.20	104	848.17	65.76	100	846.64	67.30	100	848.07	68.80
	4000	97	868.61	52.89	96	864.87	55.80	95	860.01	56.85	93	856.12	59.67	93	856.55	62.89
	10000	101	871.52	59	101	868.54	59.53	100	860.81	64.22	99	855.18	65.39	96	853.74	67.87
**TbBMD**	400	105	176.44	37.66	105	177.78	37.70	104	177.58	38.19	100	177.37	39.78	100	179.15	40.33
	4000	97	174.84	35.17	96	176.03	36.04	95	175.86	36.55	93	175.90	37.95	93	178.26	39.06
	10000	101	171.89	38.72	101	172.70	38.78	100	172.98	38.73	99	172.91	40.26	96	175.19	40.81
**TbBV/TV**	400	105	25.46	5.3	105	25.64	5.38	104	25.62	5.44	100	25.62	5.59	100	25.90	5.61
	4000	97	25.15	4.89	96	25.25	5.03	95	25.29	5.09	93	25.33	5.24	93	25.63	5.36
	10000	101	24.91	5.31	101	25.02	5.35	100	25.05	5.36	99	25.05	5.54	96	25.39	5.56
**TbTh**	400	105	0.27	0.04	105	0.27	0.04	104	0.26	0.03	100	0.27	0.03	100	0.27	0.03
	4000	97	0.26	0.03	96	0.27	0.03	95	0.26	0.03	93	0.27	0.03	93	0.27	0.03
	10000	101	0.27	0.02	101	0.27	0.03	100	0.27	0.02	99	0.27	0.03	96	0.27	0.03
**TbSp**	400	105	0.74	0.12	105	0.73	0.11	104	0.72	0.12	100	0.72	0.12	100	0.72	0.12
	4000	97	0.74	0.12	96	0.73	0.12	95	0.73	0.13	93	0.73	0.13	93	0.73	0.14
	10000	101	0.77	0.18	101	0.77	0.18	100	0.77	0.19	99	0.77	0.18	96	0.77	0.19
**TbN**	400	105	1.33	0.19	105	1.35	0.19	104	1.36	0.20	100	1.38	0.22	100	1.37	0.21
	4000	97	1.33	0.19	96	1.36	0.19	95	1.35	0.20	93	1.36	0.20	93	1.37	0.20
	10000	101	1.30	0.24	101	1.31	0.24	100	1.32	0.25	99	1.32	0.25	96	1.32	0.25
**CtTh**	400	105	1.58	0.36	105	1.58	0.36	104	1.58	0.36	100	1.56	0.33	100	1.53	0.32
	4000	97	1.66	0.32	96	1.66	0.31	95	1.65	0.31	93	1.64	0.29	93	1.59	0.28
	10000	101	1.60	0.31	101	1.60	0.31	100	1.60	0.30	99	1.58	0.29	96	1.54	0.29

**CtPo**	400	105	2.94	1.27	105	2.88	1.22	104	2.99	1.33	100	2.94	1.24	100	2.91	1.22
	4000	97	2.90	1.20	96	2.91	1.23	95	3.08	1.29	93	3.06	1.36	93	3.04	1.41
	10000	101	2.79	1.26	101	2.83	1.31	100	2.95	1.42	99	3.04	1.44	96	2.96	1.41
**DA**	400	105	1.47	0.09	105	1.46	0.09	104	1.45	0.09	100	1.44	0.09	100	1.45	0.09
	4000	97	1.45	0.09	96	1.44	0.09	95	1.44	0.09	93	1.43	0.09	93	1.43	0.09
	10000	101	1.45	0.08	101	1.45	0.08	100	1.44	0.08	99	1.44	0.09	96	1.44	0.09
**ConnD**	400	105	2.57	0.64	105	2.62	0.65	104	2.62	0.64	100	2.64	0.69	100	2.65	0.68
	4000	97	2.59	0.64	96	2.61	0.63	95	2.63	0.68	93	2.61	0.62	93	2.61	0.61
	10000	101	2.50	0.71	101	2.53	0.72	100	2.54	0.74	99	2.51	0.73	96	2.51	0.73
**SMI**	400	105	1.70	0.98	105	1.76	0.98	104	1.77	1.01	100	1.81	0.98	100	1.74	0.93
	4000	97	1.90	1.04	96	1.96	1.03	95	1.91	1.04	93	1.92	0.99	93	1.93	1.06
	10000	101	1.86	1.02	101	1.88	1.01	100	1.88	1.01	99	1.86	1.06	96	1.80	0.96
**Failure Load**	400	105	7831	2420	105	7811	2380	104	7794	2437	100	7740	2482	100	7785	2443
	4000	97	7660	2001	96	7595	2023	95	7621	2029	93	7536	2057	93	7576	2124
	10000	101	7533	2209	101	7512	2185	100	7452	2206	99	7418	2261	96	7412	2265
**Failure Load [a]**	400	105	9601	2679	105	9591	2644	104	9577	2708	100	9531	2742	100	9546	2729
	4000	97	9431	2307	96	9367	2321	95	9389	2335	93	9257	2409	93	9324	2430
	10000	101	9303	2480	101	9275	2463	100	9226	2484	99	9178	2526	96	9164	2557

Trabecular bone volume fraction (TbBV/TV, %), trabecular thickness (TbTh, mm), trabecular separation (TbSp, mm), cortical thickness (CtTh, mm), cortical perimeter (CtPm, mm), cortical pore diameter (CtPoDm, mm), degree of anisotropy (DA, unitless), connectivity density (ConnD, $1/mm^3$), structural model index (SMI, unitless). Failure load (N) is reported as unregistered (previously reported) and registered [a].

Supplementary Table 4. Raw DXA results at each time point

		**0**			**12**			**24**			**36**		
	IU	**N**	**Mean**	**SD**	**N**	**Mean**	**SD**	**N**	**Mean**	**SD**	**N**	**Mean**	**SD**
**LS aBMD**	400	90	1.183	0.172	89	1.191	0.186	87	1.187	0.187	87	1.184	0.181
	4000	79	1.202	0.161	78	1.209	0.170	76	1.214	0.173	76	1.205	0.182
	10000	87	1.188	0.176	86	1.191	0.184	85	1.186	0.186	82	1.186	0.192
**LS T-Score**	400	90	0.046	1.433	89	0.115	1.534	87	0.090	1.546	87	0.082	1.508
	4000	79	0.203	1.334	78	0.263	1.404	76	0.313	1.431	76	0.246	1.506
	10000	87	0.062	1.432	86	0.085	1.495	85	0.042	1.524	82	0.062	1.593
**TBS**	400	87	1.397	0.096	86	1.401	0.093	83	1.392	0.093	83	1.402	0.103
	4000	75	1.375	0.100	74	1.383	0.091	71	1.372	0.095	72	1.390	0.087
	10000	84	1.413	0.095	84	1.418	0.100	83	1.404	0.100	80	1.411	0.093
**FN aBMD**	400	104	0.965	0.131	103	0.961	0.128	100	0.954	0.133	99	0.953	0.134
	4000	96	0.972	0.124	95	0.975	0.130	93	0.974	0.133	93	0.967	0.134
	10000	101	0.951	0.132	100	0.954	0.135	99	0.950	0.138	95	0.943	0.140
**FN T-Score**	400	104	-0.525	0.937	103	-0.557	0.925	100	-0.603	0.955	99	-0.613	0.960
	4000	96	-0.471	0.899	95	-0.456	0.930	93	-0.467	0.952	93	-0.512	0.962
	10000	101	-0.626	0.953	100	-0.611	0.967	99	-0.627	0.993	95	-0.685	1.005
**TH aBMD**	400	104	1.022	0.138	103	1.022	0.138	100	1.016	0.141	99	1.015	0.141
	4000	96	1.035	0.140	95	1.040	0.152	93	1.035	0.150	93	1.030	0.149
	10000	101	1.008	0.138	100	1.009	0.139	99	1.006	0.141	95	1.001	0.139
**TH T-Score**	400	104	0.116	1.093	103	0.111	1.099	100	0.072	1.117	99	0.062	1.122
	4000	96	0.224	1.109	95	0.256	1.200	93	0.223	1.190	93	0.180	1.183
	10000	101	0.000	1.097	100	0.013	1.103	99	-0.012	1.113	95	-0.056	1.108
**UD aBMD**	400	105	0.482	0.098	104	0.484	0.098	100	0.483	0.101	100	0.481	0.100
	4000	97	0.476	0.098	96	0.478	0.101	94	0.475	0.101	93	0.473	0.102
	10000	101	0.471	0.103	100	0.468	0.102	99	0.460	0.104	96	0.457	0.106

	400	105	-0.341	1.678	104	-0.307	1.678	100	-0.339	1.727	100	-0.388	1.723
**UD T-Score**	4000	97	-0.393	1.746	96	-0.36	1.803	94	-0.432	1.803	93	-0.471	1.829
	10000	101	-0.515	1.767	100	-0.596	1.751	99	-0.751	1.791	96	-0.836	1.836
	400	105	0.906	0.138	104	0.909	0.140	100	0.909	0.143	100	0.908	0.145
**33% aBMD**	4000	97	0.908	0.130	96	0.902	0.133	94	0.908	0.137	93	0.906	0.139
	10000	101	0.909	0.128	100	0.897	0.133	99	0.896	0.138	96	0.894	0.146
	400	105	-0.397	1.142	104	-0.378	1.167	100	-0.376	1.205	100	-0.389	1.200
**33% T-Score**	4000	97	-0.305	1.053	96	-0.388	1.082	94	-0.316	1.120	93	-0.349	1.143
	10000	101	-0.309	0.969	100	-0.447	1.022	99	-0.463	1.071	96	-0.502	1.159

Areal bone mineral density (aBMD, g/cm^2), lumbar spine (LS), trabecular bone score (TBS), femoral neck (FN), total hip (TH), ultra-distal (UD).
